# Electroosmotic Flows of Power-Law Fluids with Asymmetric Electrochemical Boundary Conditions in a Rectangular Microchannel

**DOI:** 10.3390/mi8050165

**Published:** 2017-05-20

**Authors:** WooSeok Choi, Sungchan Yun, Du-Soon Choi

**Affiliations:** Department of Mechanical Engineering, Korea National University of Transportation, Chungju 27469, Korea; w.choi@ut.ac.kr (W.C.); syun@ut.ac.kr (S.Y.)

**Keywords:** electroosmosis, power-law fluid, zeta potential, microchannel

## Abstract

In this paper, a systematic study of a fully developed electroosmotic flow of power-law fluids in a rectangular microchannel bounded by walls with different zeta potentials is described. Because the upper and lower layers of most microchannels are made of different materials, it is necessary to study the flow characteristics for cases in which the microchannels have different zeta potentials at each wall. The electrical potential and momentum equations were solved numerically using a finite element analysis. The velocity profiles and flow rates were studied parametrically by varying the fluid behavior index, channel aspect ratio, and electrochemical properties of the liquid and the bounding walls. The calculated volumetric flow rates in a rectangular microchannel were compared with those between two infinite parallel plates.

## 1. Introduction

Electroosmotic flow (EOF) is one of the most important techniques in a microfluidic system because conventional pressure-driven flows are inefficient owing to a high surface-to-volume ratio at the microscale. The mechanism and underlying physics of an EOF are now considered rather classical materials, as described in many different textbooks [1,2,3,4]. Most studies on EOFs have assumed that the medium in the microchannel is a Newtonian fluid, which is a rational consideration because most electrolytes or buffer solutions used in microfluidic devices are Newtonian. However, biological fluids such as blood, saliva, and DNA solutions, which are frequently used in microfluidic devices such as biochips, are non-Newtonian in nature. Because non-Newtonian fluids show different flow behaviors than Newtonian fluids, it is suitable for a non-Newtonian model to predict the appropriate EOF [5,6,7,8,9,10,11,12].

Among the various models for non-Newtonian fluids, the power-law model has been the most chosen rheological model for EOFs occurring in a microchannel, owing to its simplicity and adequateness in terms of the flow behavior [13,14]. Using the power law, Das and Chakraborty analyzed the EOF behavior of blood samples as functions of the blood concentration [7]. Zhao et al. analyzed the behavior of an EOF in a slit channel using the power-law, and solved the analytical expressions for the shear stress, dynamic viscosity, and velocity distribution [15]. In addition, they obtained a general Smoluchowski slip velocity using the Carreau model [16]. Bharti et al. investigated the electroviscous effects in a steady, fully developed flow of a power-law fluid through a cylindrical microchannel using the finite difference method [13]. Tang et al. numerically studied a non-Newtonian power-law fluid in a microchannel when applying the lattice Boltzmann methodology [17]. Berli reported the theoretical expressions of the flow rate and output pressure of the electrokinetic pumping of non-Newtonian fluids through cylindrical and slit microchannels [18]. Vasu and De solved the EOF behavior of power-law fluids in a slit microchannel at a high zeta potential without applying a linear Debye-Hückel approximation [19]. Babaie et al. reported the EOF of a power-law fluid in a slit microchannel by combining the pressure gradient in the channel [20]. Ng and Qi presented an analytical model for the EOF of a power-law fluid through a slit channel while gradually varying the channel height and wall potential [14]. In addition, some efforts have focused on viscoelastic fluids in two-dimensional microchannels under the combined influence of electroosmotic and pressure gradient forces. Afonso et al. investigated the steady-state slip flow with symmetric or asymmetric zeta potentials at the walls [21]. Wang et al. investigated the unsteady slip flow with asymmetric zeta potentials at the walls [22].

In most lab-on-a-chip systems, the cross-sections of the microchannels, made using modern micromachining technology, are close to a rectangular shape [23,24]. However, in studies on the EOF of a non-Newtonian fluid, there has been very little research on the flow inside a rectangular channel as compared to research on circular and parallel plate microchannels. Park and Lee obtained the volumetric flow rate using the Helmholtz-Smoluchowski velocity for viscoelastic fluids while applying the Phan-Thien Tannar model with and without a pressure gradient [12]. Vakili et al. reported a hydrodynamically fully developed EOF of a power-law fluid in a rectangular channel with a changing aspect ratio using a numerical solution through a finite difference procedure [24]. Zhao et al. studied the transient EOF of power-law fluids in a rectangular microchannel driven by three modes of an electric field, i.e., a direct current (DC) electric field, an alternating current (AC) electric field, and a combined AC and DC electric field using a finite element method [25]. The above studies are based on a symmetric rectangular channel structure, assuming identical zeta potentials of the walls encompassing the microchannel. However, there are many cases that call for using different materials to fabricate a useful microchannel. A common example is a microchannel made with a polydimethylsiloxane (PDMS) top and a silicon dioxide (glass) bottom [26]. Datta et al. obtained analytical solutions in the form of a Fourier series for EOF in a rectangular channel with variable wall zeta-potential for Newtonian fluids [27], however, EOF in a rectangular channel with asymmetric zeta potential for non-Newtonian fluids has not yet been reported.

In the present study, we generalize previous studies on power-law fluids and introduce bounding walls with different zeta potentials and different aspect ratios of the rectangular channel. Herein, we report the characteristics of an EOF by comparing and looking into the velocity profiles and volumetric flow rates in rectangular channels and between infinite parallel plates.

## 2. Mathematical Formulation

**Figure 1:**
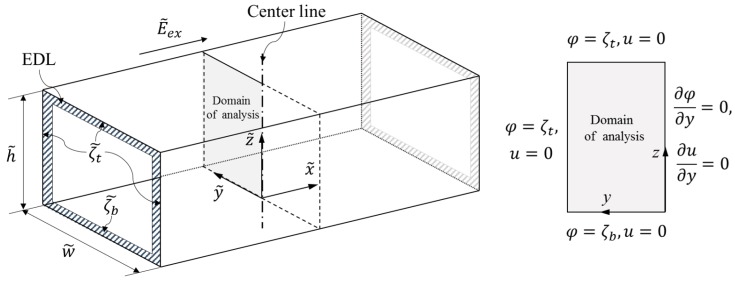
Schematic diagram of electroosmotic flow in a rectangular microchannel and boundary conditions.

We consider a fully developed steady-state electroosmotic flow, as shown in Figure 1, where a constant direct current (DC) of electric field ${\tilde{E}}_{ex}$ is applied to a non-Newtonian fluid with a constant density $\tilde{\rho }$ and electric permittivity $\tilde{\epsilon }$ confined to a rectangular channel, where $\tilde{w}$ and $\tilde{h}$ indicate the width and height, respectively. Each boundary bears an electric charge upon contact with a fluid. We assume that the top and side walls are quantified based on zeta potential ${\tilde{\zeta }}_{t}$, and that the bottom wall is quantified based on zeta potential ${\tilde{\zeta }}_{b}$. Thin nano-scale regions with excess ions, called an electrical double layer (EDL), are formed adjacent to each boundary, as indicated by the hatched area in the figure, and are labeled as exaggerated EDL for clarity. The flow is caused by the electrostatic reaction between an external electric field ${\tilde{E}}_{ex}$ and an excessive amount of ions in the EDLs, which will drag the entire fluid through viscous dissipation.

We describe this EOF theoretically based on a system of equations nondimensionalized using $\tilde{h}$, ${\tilde{E}}_{ex}\tilde{h}$, ${\tilde{u}}_{s}=-\epsilon {\tilde{\zeta }}_{t}{\tilde{E}}_{ex}/{\tilde{\mu }}_{0}$, and $\tilde{t}=\tilde{h}/{\tilde{u}}_{s}$ as the characteristic length, electric potential, velocity, and time, respectively, where ${\tilde{u}}_{s}$ and ${\tilde{\mu }}_{0}$ are the conventional Smoluchowski velocity and dynamic viscosity of Newtonian fluids, and the superscript tilde denotes dimensional form. The velocity field of a fluid in a rectangular channel is governed by the dimensionless continuity and Cauchy momentum equations, which are given as follows:

(1) ∇·u=0,

(2) ReDuDt=−∇p+∇·τ+fe,

where u is the velocity vector, p is the pressure, τ is the stress tensor, and $f_{e}$ is the body force. The dimensionless stress tensor τ can be expressed based on the strain rate tensor, D, as:

(3) τ=2μ(D)D,

where $D=\left\{\nabla u+{\left(\nabla u\right)}^{T}\right\}/2$ and μ(D) is the effective viscosity. The effective viscosity for the power law fluid is given by:

(4) $$\mu \left(D\right)=m{\left(\sqrt{2D:D}\;\right)}^{n-1},$$

where m is the flow consistency index, which is nondimensionalized using ${\tilde{\mu }}_{0}{\tilde{h}}^{n-1}/{\tilde{u}}_{s}^{n-1}$, and n is the flow behavior index. We consider the unidirectional flow that can be represented as $u=u\left(y,z\right)e_{x}$, where u is the x-component of velocity and $e_{x}$ is the unit vector along the *x*-direction. For the EOF, the only driving force is generated through the interaction between the external electric field ${\tilde{E}}_{ex}$ and the charge density in the EDL region. A dimensionless body force in Equation (2) can be obtained by introducing dimensionless zeta potential based on the top and side walls $E_{R}={\tilde{\zeta }}_{t}/\left({\tilde{E}}_{ex}\tilde{h}\right)$ to the Cauchy momentum equation, which gives us $f_{e}=\nabla^{2}\phi \nabla \phi /E_{R}$: the details of the dimensionless form are described in Appendix A. The total electric potential can be represented as ϕ(x,y,z)=−x+φ(y,z), where −x and φ(y,z) are the electric potential owing to an external electric field and the zeta potentials, respectively. If we limit the present analysis to microchannels where the Debye length ${\tilde{\lambda }}_{D}$ is much smaller than the height $\tilde{h}$ of the channel, the electric potential owing to the zeta potential can be described through the Poisson-Boltzmann equation, which can be linearized using a Debye-Hückel approximation as:

(5) $$\nabla^{2}\varphi =\varphi /L_{D}^{2},$$

where $L_{D}={\tilde{\lambda }}_{D}/\tilde{h}$ is the nondimensional Debye length. Here, the Debye length $\left({\tilde{\lambda }}_{D}\right)$, a measure of the EDL thickness, is expressed as ${\tilde{\lambda }}_{D}=\sqrt{\tilde{\epsilon }{\tilde{k}}_{B}\tilde{T}/2{\tilde{e}}^{2}z^{2}{\tilde{N}}_{A}{\tilde{c}}_{0}}$ for an aqueous solution of a symmetrical electrolyte, where ${\tilde{k}}_{B}$ is a Boltzmann constant, $\tilde{T}$ is the absolute temperature, $\tilde{e}$ is the elementary charge density, z is the charge number of ions, ${\tilde{N}}_{A}$ is the Avogadro’s number, and ${\tilde{c}}_{0}$ is the mole concentration (mol/m^3^).

In the present analysis, both EDL potential field and electroosmotic flow field are solved in the partial differential equation (PDE) module of finite element numerical analysis package COMSOL Multiphysics 5.1. In our work, a PDE governing the EDL potential (Equation (5)) and a PDE governing electroosmotic flow field (Equation (2)) are both constructed from the general form of PDE in COMSOL, which is based on the numerical method used in the work by Zhao et al. [15]. Through the body force term $f_{e}=\nabla^{2}\phi \nabla \phi /E_{R}$, these two PDEs are coupled together. A no-slip condition was applied to each wall, and a symmetric condition was applied to the symmetric surface, as shown in Figure 1.

The parameters used in this study are the zeta potential ratio $\left(Z_{R}={\tilde{\zeta }}_{b}/{\tilde{\zeta }}_{t}\right)$, ranging from −1 to 2; the fluid behavior index (n), ranging from 0.8 to 1.2; the channel aspect ratio $\left(A_{R}=\tilde{w}/\tilde{h}\right)$, ranging from 0.5 to 5; and the dimensionless Debye length $\left(L_{D}\right)$, ranging from 0.005 to 0.01.

## 3. Results

In the calculations, the following parameters and constants are used: the external electric field, ${\tilde{E}}_{ex}=10$ kV; the relative permittivity, $\epsilon_{r}=80$; the vacuum permittivity, ${\tilde{\epsilon }}_{0}=8.85\times 10^{-12}\;$F/m; the absolute temperature, $\tilde{T}=300$ K; the bottom wall zeta potential, ${\tilde{\zeta }}_{b}=-60$ mV; the Boltzmann constant, ${\tilde{k}}_{b}=1.38\times 10^{-23}$ J/K; the valence of ions, z=1; and the dynamic viscosity of Newtonian fluid, ${\tilde{\mu }}_{0}=1.12\times 10^{-3}\;N\cdot s/m^{2}$. In this study, we neglected the effect of pressure gradient and the electroviscous effect.

**Figure 2:**
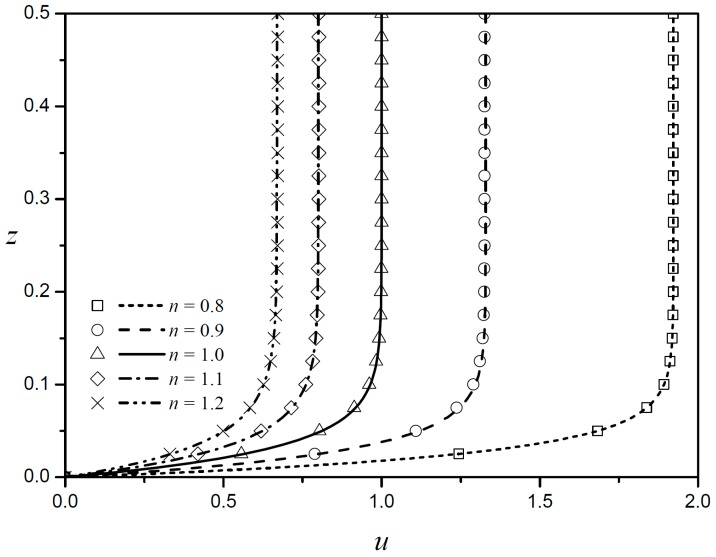
Velocity profiles obtained by Zhao et al. [15] against the present results for five different fluid behavior indices for $L_{D}$ = 0.03. Symbols, Zhao et al.; lines, present results.

When the width and height of the rectangular channel are sufficiently large and the zeta potentials of all boundaries are the same, the velocity profiles along the centerline of the channel should coincide with the analytical results derived by Zhao et al. [15]. Figure 2 shows such cases for five different flow behavior indices. The present results (lines) and those by Zhao et al. [15] (symbols) are shown to coincide exactly for all five cases shown. Because the top and bottom are symmetrical, only half of the velocity profiles are displayed. For a purely viscous Newtonian fluid (n=1.0), a characteristic plug-flow type EOF with a Helmoholtz-Smoluchowski is observed in the core region, with large velocity gradients in the thin boundary regions. Under the same electroosmotic conditions, as the flow behavior index decreases (n<1), the flow velocity in the core region increases conspicuously, and then decreases as the flow behavior index increases (n>1). This is because shear-thickening fluids (n>1) require larger shear stresses than Newtonian fluids as the velocity gradient increases. As a result, shear-thickening fluids have lower velocity gradients than Newtonian fluids under the same conditions.

**Figure 3:**
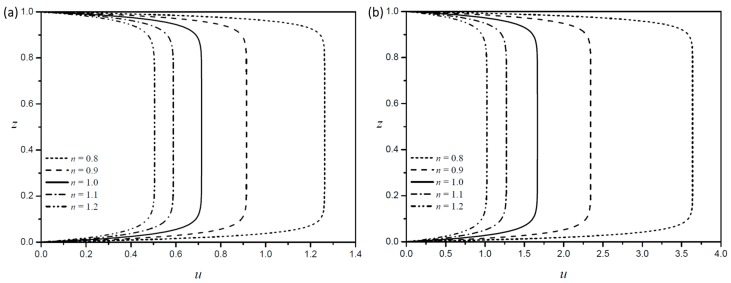
Velocity profiles along the centerline with $A_{R}=1$ and $L_{D}=0.03$ according to the consistency index of (**a**) a shear-thinning fluid (n=0.8) and (**b**) a shear-thickening fluid (n=1.2).

Figure 3a,b show the velocity profiles along the centerline of the square channel with identical zeta potential for five different fluid consistency indices (m) with a fixed Debye length ($L_{D}=0.03$). Velocity distributions for a shear-thinning fluid (n=0.8) are plotted in Figure 3a, and those for a shear-thickening fluid (n=1.2) are plotted in Figure 3b. The results show that the velocity distribution has the same tendency as the fluid consistency index (m) increases, which can also be seen in the results of Zhao et al. [15]. Therefore, we kept the fluid consistency index m=1 in this paper.

**Figure 4:**
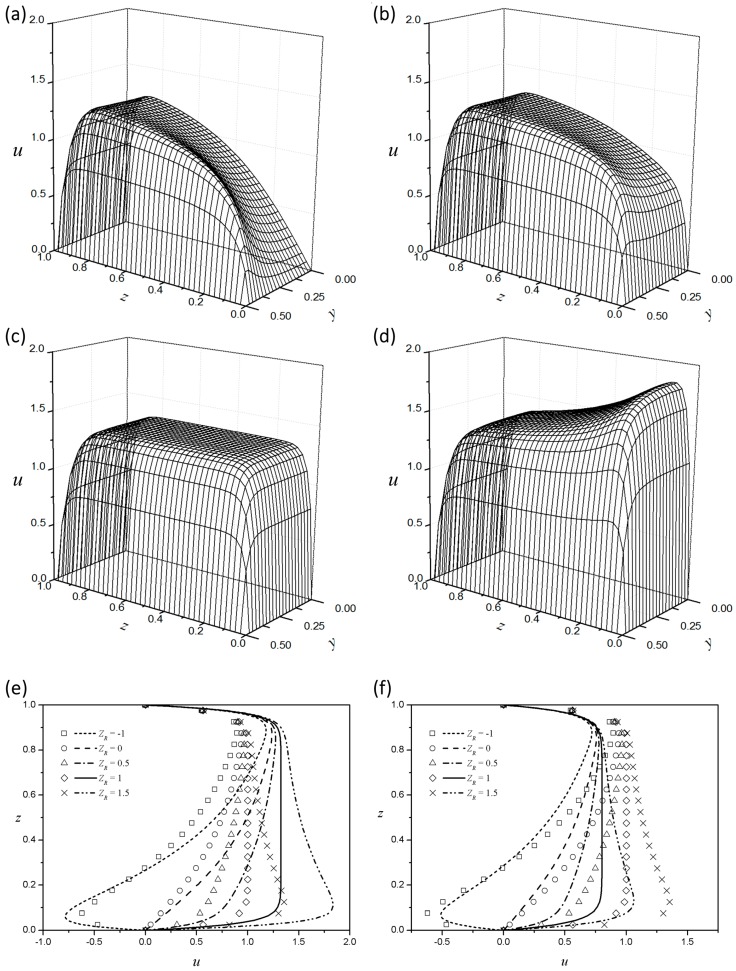
Velocity distribution in a square microchannel with $L_{D}=0.03$ and n=0.9 for different zeta potential ratios: (**a**) $Z_{R}=0$; (**b**) $Z_{R}=0.5$; (**c**) $Z_{R}=1.0$; and (**d**) $Z_{R}=1.5$. Velocity profiles along the centerline with $A_{R}=1$ and $L_{D}=0.03$ according to the zeta potential ratio of (**d**) a shear-thinning fluid (n=0.9) and (**e**) a shear-thickening fluid (n=1.1) (symbols, Newtonian fluid; n=1.0).

When the top and bottom boundaries are of different materials, and thus of different zeta potentials $\left(Z_{R}\neq 1\right)$, the symmetry of the EOF is broken. Various velocity profiles that differ from those above will then be possible. Figure 4a–d show the velocity distributions in a rectangular channel for four different zeta potential ratios with a fixed aspect ratio $\left(A_{R}=1\right)$, Debye length $\left(L_{D}=0.03\right)$, and behavior index (n=0.9). Figure 4e,f show the velocity profiles along the centerline according to the zeta potential ratio for a flow index of 0.9 (Figure 4e) and 1.1 (Figure 4f), respectively. The velocity profile of a Newtonian fluid is also added for comparison. In addition, the symmetry case of $Z_{R}=1$ is included in the figure as a reference. If the zeta potential at the bottom boundary is larger than those at the other boundaries $\left(Z_{R}>1\right)$, the entire EOF is enhanced, with the center portion near the bottom wall being more pronounced. If the zeta potential at the bottom boundary is lower than those at the other boundaries and has the same sense $\left(0<Z_{R}<1\right)$, the flow at the center portion near the bottom wall is less pronounced. If the bottom boundary is electrochemically inert $\left(Z_{R}=0\right)$, the EDL adjacent to the bottom boundary does not contribute to the EOF. The flow is thus generated from viscous dissipation originating from the top and side boundaries. When the zeta potential at the bottom boundary has the opposite sense $\left(Z_{R}<0\right)$, the Maxwell stress at the bottom EDL will generate an EOF in the opposite (−x) direction, as shown in the figure. The smaller the behavior index of the power-law fluid, the greater the velocity gradient near the EDL. As described above, the entire EOF of the shear-thinning fluid is enhanced compared with the Newtonian case.

**Figure 5:**
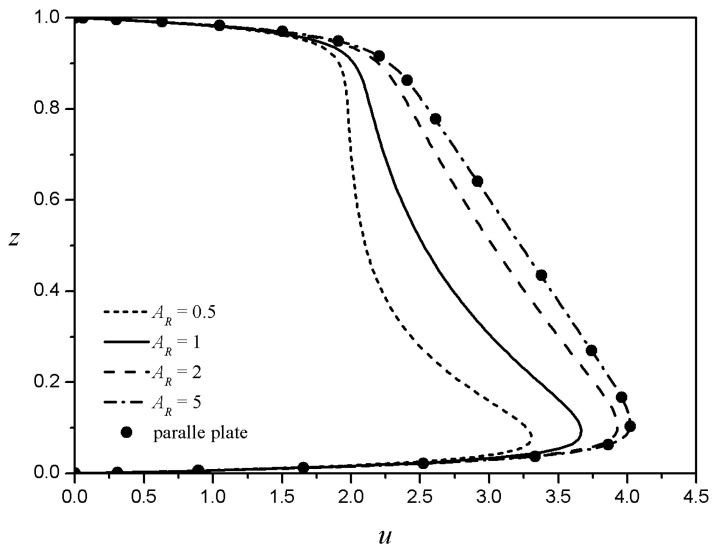
Velocity profiles according to the channel aspect ratio at $L_{D}=0.03$, $Z_{R}=2$, and n=0.8.

**Figure 6:**
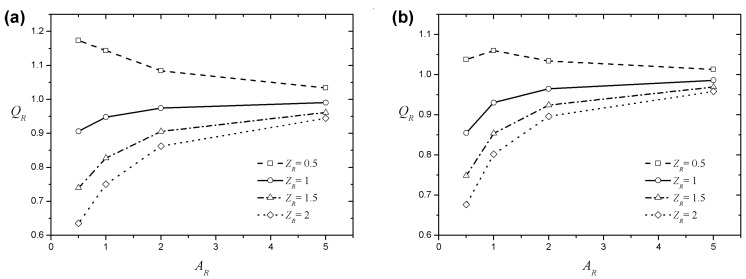
The flow rate ratio according to the channel aspect ratio and zeta potential ratio at $L_{D}=0.03$: (**a**) n=0.8 and (**b**) n=1.2.

The flow enhancement should also vary depending on the aspect ratio of the rectangular channel. Figure 5 shows the velocity profile along the center of a rectangular channel for four different aspect ratios with a fixed zeta potential ratio $\left(Z_{R}=2\right)$, Debye length $\left(L_{D}=0.03\right)$, and behavior index (n=0.8). The symbols indicate the EOF generated between the infinite parallel plates, whereas the line shows the velocity profile in the rectangular channel. If there are no side walls (an EOF is generated between the parallel plates), the velocity profile at the centerline between the top and bottom walls is linear outside of the EDL. Because the velocity induced by the zeta potential of the side wall is different from the velocity at the centerline, a velocity gradient for the width direction is created, and the velocity of the middle part becomes less pronounced, with a bent velocity profile, as shown in the figure. When $Z_{R}<1$, the velocity profile will be bent in the opposite direction. When the channel is widened, the velocity gradient is lowered, the influence of the velocity induced by the side wall will decrease, and the velocity profile of the core portion becomes closer to a linear state. The flow enhancement can be examined more quantitatively by studying the flow rate, which can be obtained through an integration of the velocity in the cross-section of the microchannel. Here, the flow rate ratio $\left(Q_{R}\right)$ is used to examine the flow characteristics in a rectangular channel. The flow rate ratio can be obtained by dividing the volumetric flow rate per unit area in a rectangular microchannel by that between infinite parallel plates without side walls. Figure 6a,b show the relationship between the ratio of the flow rate and the aspect ratio of the microchannel for various zeta potential ratios when $L_{D}=0.03$. In Figure 6a, $Q_{R}$ is plotted against the aspect ratio for a shear-thinning fluid (n=0.8), whereas $Q_{R}$ of a shear-thickening fluid (n=1.2) is plotted in Figure 6b. The shear-thinning and -thickening fluids tend to be quite similar. In both cases, they are affected by the side walls in the same way although there is a difference in degree. When the zeta potential of the bottom wall is larger than that of the side walls $\left(Z_{R}>1\right)$, $Q_{R}$ always has a value of less than unity because the velocity of the middle portion of the channel is less pronounced by the influence of the side wall. Intuitively, as $A_{R}$ increases, $Q_{R}$ approaches unity, which means that the wider the channel is, the less it is affected by the side wall. As shown in Figure 6b, $Q_{R}$ at $A_{R}=0.5$ is smaller than at $A_{R}=1$ when $Z_{R}=0.5$. This is because the width of the channel becomes narrower, and the velocity gradient section overlaps and is not sufficiently influenced by the velocity increase from the side walls.

**Figure 7:**
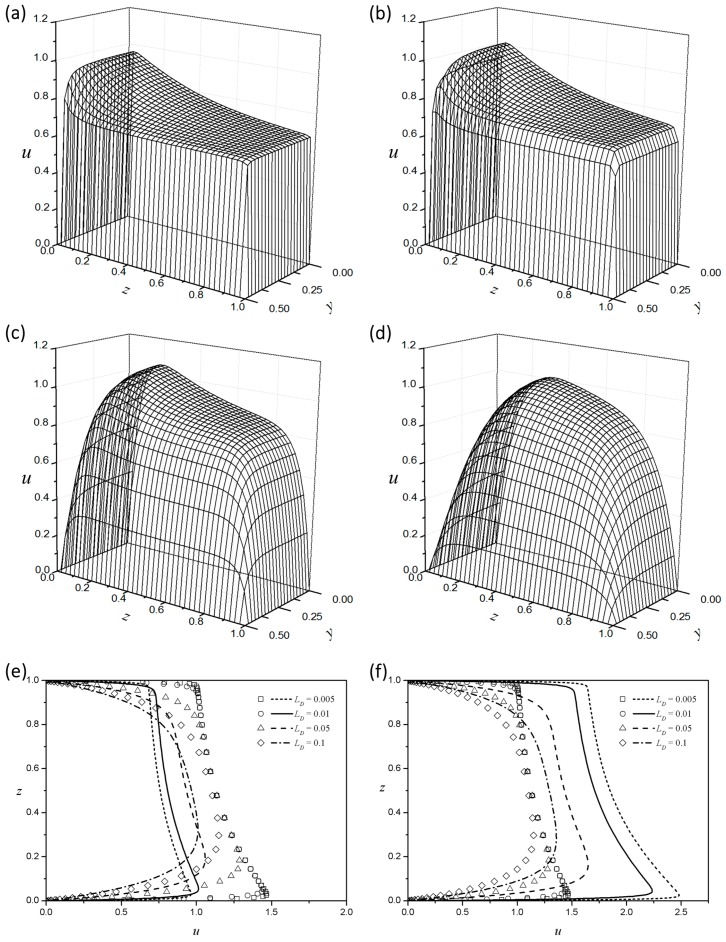
Velocity distribution at different values of Debye length for n=1.1 and $Z_{R}=1.5$: (**a**) $L_{D}=0.005$; (**b**) $L_{D}=0.01$; (**c**) $L_{D}=0.05$; and (**d**) $L_{D}=0.1$. Velocity profiles along the centerline according to different Debye lengths for $Z_{R}=1.5$ and $A_{R}=1$. Symbols, Newtonian fluid: (**e**) shear-thinning (n=0.9) and (**f**) shear-thickening (n=1.1) fluids.

**Figure 8:**
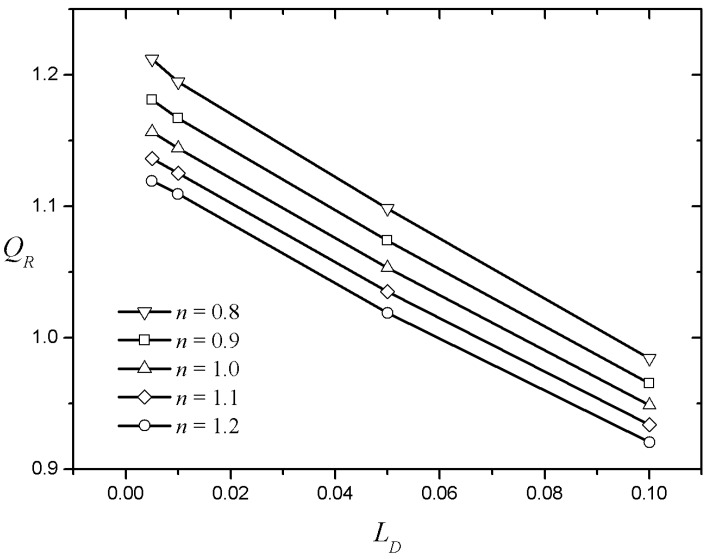
The flow rate ratio according to the Debye length for different behavior indices.

Increasing the Debye length $L_{D}$ turns the plug-flow type velocity profiles into a more parabolic shape, as shown in Figure 7. Figure 7a–d show the velocity distributions in a rectangular channel for four different dimensionless Debye lengths $\left(L_{D}\right)$ for a shear-thickening fluid (n=1.1) at $Z_{R}=1.5$. The velocity profiles of the Newtonian fluids are almost identical around the center, irrespective of the Debye length. On the other hand, the power-law fluid flow enhancement will thus appear with a decrease in $L_{D}$. The shear-thinning fluid increases the core velocity as the EDL thickness decreases (Figure 7e), and the shear-thickening fluid decreases the core velocity (Figure 7f). In Figure 8, $Q_{R}$ is plotted against $L_{D}$ for five different index values with fixed aspect $\left(A_{R}=1\right)$ and zeta potential $\left(Z_{R}=0.5\right)$ ratios. Regardless of the index value, $Q_{R}$ tends to decrease almost linearly as the Debye length increases, because the flow rate decreases as the flow enhancement area around the side wall widens. As the index increases, the $Q_{R}$ graph according to the Debye length decreases in parallel. Interestingly, the value of $Q_{R}$ decreases from more than unity to less than unity for all cases as the Debye length decreases. This means that, if the Debye length is small, the flow rate per unit area of an EOF in a square microchannel is larger than that of an EOF generated between the infinite parallel plates under the same conditions. Intuitively, $Q_{R}$ is always considered to be larger than unity when $Z_{R}<1$ because the side walls with a relatively large zeta potential increase the flow rate. However, if the Debye length is sufficiently large, the velocity increment caused by the EDL near the side walls will overlap before becoming fully developed. Therefore, the influence of the flow rate decreasing by the no-slip condition of the wall becomes larger than the effect of velocity enhancement owing to free charges in the EDL of the side walls.

## 4. Conclusions

We studied electroosmotic flows of non-Newtonian fluids with an asymmetric boundary in a rectangular microchannel by applying the power-law method. Zeta potentials on the top and bottom boundaries are allowed to assume mutually different values, and thus they can be made up of different materials. Based on numerical simulations, the fluid flow and electric potential distribution, coupled through the hydrodynamic and Maxwell stress contributions, were analyzed based on the conservation laws, including the momentum and linearized Poisson-Boltzmann equation.

Flow enhancement of a power-law fluid was characterized in terms of the ratio of zeta potential, behavior index, aspect ratio of the channel, and Debye length. The velocity profiles of the power-law fluids show a more enhanced flow as the behavior index decreases, corresponding to the shear-thinning hydrodynamic features. In addition, it is apparent that an increase in the flow rate gives rise to a higher $A_{R}$ and lower $Z_{R}$ when the zeta potential of the bottom is substantial $\left(Z_{R}>1\right)$. Power-law fluids in the presence of side walls consistently have flow rates beyond the EOF generated between infinite parallel plates when the zeta potential at the top is substantial $\left(0<Z_{R}<1\right)$. As the Debye length increases, the flow rates of the power-law fluids decrease almost linearly. It can be concluded from the results that, depending on the Debye length and difference in zeta potential, the flow rate in a rectangular microchannel may be greater or less than the flow rate between two parallel infinite plates under identical conditions. This work can help in understanding the hydrodynamics of rheological fluids, such as biological fluids including blood, saliva, and DNA solutions, in a typical channel employed in a lab-on-a-chip system.

## Appendix A

The Cauchy momentum equation can be non-dimensionalized in the following ways:

(A1) $$\frac{\tilde{\rho }\left(d\tilde{u}\right)}{d\tilde{t}}=-\tilde{\nabla }\tilde{p}+\tilde{\nabla }\cdot \tilde{\tau }+{\tilde{f}}_{e}$$

(A2) $$\tilde{\rho }\frac{{\tilde{u}}_{s}^{2}}{\tilde{h}}\frac{du}{dt}=-\frac{{\tilde{\mu }}_{0}{\tilde{u}}_{s}}{{\tilde{h}}^{2}}\nabla p+\frac{{\tilde{\mu }}_{0}{\tilde{u}}_{s}}{{\tilde{h}}^{2}}\nabla \cdot \tau +\frac{\tilde{\epsilon }{\tilde{E}}_{ex}^{2}}{\tilde{h}}\nabla^{2}\phi \nabla \phi$$

(A3) $$\frac{\tilde{\rho }{\tilde{u}}_{s}\tilde{h}}{{\tilde{\mu }}_{0}}\frac{du}{dt}=-\nabla p+\nabla \cdot \tau +\frac{\tilde{\epsilon }{\tilde{E}}_{ex}{\tilde{\zeta }}_{t}}{{\tilde{\mu }}_{0}}\frac{1}{{\tilde{u}}_{s}}\frac{{\tilde{E}}_{ex}\tilde{h}}{{\tilde{\zeta }}_{t}}\nabla^{2}\phi \nabla \phi$$

(A4) Redudt=−∇p+∇·τ+1ER∇2ϕ∇ϕ

where the superscript tilde denotes dimensional form.
